# Bibliometrics and Visualization Analysis of Three Obligate Organohalide Respiring Bacteria Genera: A Systematic Review

**DOI:** 10.3390/microorganisms13071668

**Published:** 2025-07-16

**Authors:** Lisi Jiang, Zirui Yu, Jiaqi Qu, Xiaohan Xu, Zirui Liu, Wenyuan Li, Yang Zhang

**Affiliations:** College of Life Science, Shenyang Normal University, Shenyang 110034, China; jianglisi@synu.edu.cn (L.J.); 15542072852@163.com (Z.Y.); qjq_050309@163.com (J.Q.); xxh_101602@163.com (X.X.); 13332191531@163.com (Z.L.); lixiaohan20040106@163.com (W.L.)

**Keywords:** organohalide-respiring bacteria, reductive dechlorination, bibliometric

## Abstract

Organohalide-respiring bacteria (OHRB) facilitate the reductive dehalogenation of toxic halogenated compounds in the environment, which supports their growth and proliferation. Research conducted on OHRB has achieved notable advancements. However, given the intricacy of the ecosystem and the methodologies employed for microbial isolation, numerous constraints persist. Further exploration is imperative to elucidate the physiological characteristics, ecological functions, and technological applications of OHRB. This study aimed to evaluate the outcomes and insights of prior research via a bibliometric analysis of three obligate OHRB genera—*Dehalococcoides*, *Dehalobacter*, and *Dehalogenimonas*—over a three-decade period from 1994 to 2024, based on the Web of Science (WOS) database. The results show that research on these three bacterial genera has advanced in sequence since the initiation of studies in this field. The research area encompasses the identification and isolation of novel OHRB species, the gene sequencing of related enzymes, and the role of microorganisms in the remediation of environmental pollutants, reflecting a gradual transition from individual investigations of OHRB to the applications of microorganisms in remediating complex environmental pollution. This study systematically reviewed the past research history of this field and conducted an in-depth analysis of research hotspots. The integration of this analysis with technological development trends and practical application requirements provides a theoretical basis and innovative concepts for future research directions in the field of ecological environment restoration.

## 1. Introduction

Organohalides are extensively utilized in industrial, agricultural, and domestic contexts; however, their inherent stability and tendency to bioaccumulate have resulted in pervasive global environmental contamination [1,2,3]. The discovery of OHRB enabled bioremediation at sites contaminated by chlorinated compounds and drew a significant number of researchers to study the process [4]. Bioremediation, which employs organohalide-respiring bacteria (OHRB) under anaerobic conditions to reduce organohalides and thereby degrade organohalogen compounds, represents an environmentally sustainable approach to addressing this issue. OHRB utilize organohalides as terminal electron acceptors, facilitating reductive dehalogenation reactions through electron transfer mechanisms. This process cleaves carbon–halogen bonds, providing both metabolic energy for bacterial growth and a means of remediating contaminated sites [5]. Furthermore, the activity of OHRB contributes to natural halogen cycling processes [6]. OHRB can be categorized into two groups: obligate and non-obligate, with the primary distinction being whether organohalogen respiration serves as their sole mode of energy metabolism [7]. Among obligate organohalide-respiring bacteria, *Dehalococcoides*, *Dehalobacter*, and *Dehalogenimonas* (hereafter referred to as *Dhc*, *Dhb*, and *Dhgm*, respectively) are recognized as key versatile strains due to their diverse dehalogenase systems, which can degrade a wide range of structural organohalides [8]. Consequently, this study focuses primarily on these three bacterial genera.

A substantial body of research has previously been conducted on organohalide-respiring bacteria, encompassing a broad spectrum of disciplines. Researchers have studied genes and enzymes related to dehalogenation metabolism in organohalogen-respiring bacteria at the molecular level. These studies have employed genome sequencing, proteomic analysis, and other technologies based on ecogenomics. The objective of these studies is to identify bacteria and enzymes, and to analyze their structure, function, and mechanism of action [9,10,11,12,13,14]. At the applied engineering and ecosystem levels, the research is centered on issues such as the microbial remediation of chlorinated contaminated sites, as well as the study of the effects of various factors in the ecological environment on the remediation efficacy of organohalide-respiring bacteria [15,16,17]. At the population level, the evolutionary history of populations was traced through systematic analyses for the construction of evolutionary trees, and the mechanisms of inter-species interactions were investigated [18]. Despite the noteworthy advancements in OHRB research, the intricacies of the ecosystem and the prevailing methodologies for microbial isolation impose considerable constraints. Consequently, there is an imperative for further exploration into the physiological properties, ecological performance, and technological applications of organohalide-respiring bacteria.

Therefore, the present study relies on bibliometrics and visual analysis to organize and analyze the relevant literature in the Web of Science database. In addition, the study comprehensively analyzes the core indexes, such as publication volume, citations country, author, journal, and keywords. The objective of this study is to methodically categorize the development of OHRB, identify emerging research frontiers and existing gaps in the field, refine the key findings from each research level, predict future trends, provide guidance for subsequent research, enhance the theoretical framework, and support environmental remediation and related fields. The ultimate goal is to contribute to the remediation of global ecological and environmental pollution.

## 2. Materials and Methods

### 2.1. Data Source and Retrieval

**Figure 1:**
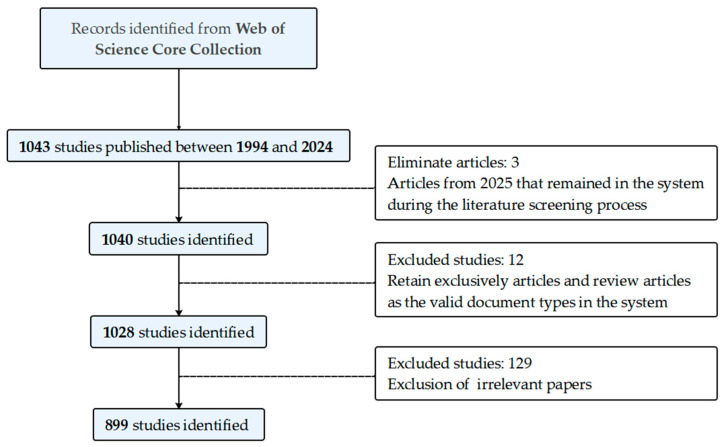
Screening flowchart. Comprehensive data screening procedure.

This study was written in accordance with the Preferred Reporting Items for Systematic Reviews and Meta-Analyses (PRISMA) guidelines (Supplementary Figure S1) [19] and was pre-registered with the Open Science Framework Registries (https://doi.org/10.17605/OSF.IO/Q5RCN; accessed on 11 July 2025). The Web of Science (WOS) is a high-quality, authoritative academic database with multiple citations. It has been recognized and accepted by most scholars as a comprehensive platform used by various researchers to retrieve articles and data. Therefore, the articles collected in this study are all from the WOS database, specifically including one major citation index. In order to circumvent the potential for bias resulting from the daily updating of the database, all screening procedures were concluded prior to 15:56 on 8 April 2025. The time frame encompassing the years from 1994 to 2024 was designated as the study period, with the understanding that papers published in 2025 would not be fully complete at the time of analysis. Data screening was carried out collaboratively by five authors. Specifically, authors were paired into two groups, with each group independently screening data and conducting comparative analyses of discrepancies. Should ambiguities arise during the process, the fifth author served as the arbiter, tasked with making final determinations on data inclusion or exclusion. The search formula employed was as follows: TS = ((“organohalide-respiring bacteria “OR” dehalogenation “OR” reductive dehalogenation “OR” reductive dehalogenase “OR” organohalide respiration “OR” dehalorespiration “OR” halorespiration”) and (“*Dehalogenimonas* “OR” *Dehalococcoides* “OR” *Dehalobacter*”)). The search terms were filtered individually, and a total of 899 documents were obtained. After a thorough review, 899 valid documents were exported as plain text files in the “full record and cited references” format for subsequent analysis (Figure 1).

### 2.2. Statistical Methods

Citespace is a Java application that combines information visualization methods, bibliometrics, and data mining algorithms. These were developed by Chaomei Chen’s team for the analysis of past studies and research hotspots, emerging trends in the field of knowledge. In this study, Citespace (CiteSpace.6.4.R1) was employed to generate the annual publication volume of papers, the keyword timeline graphs, and the keyword emergence graphs [20,21].

VOSviewer is a computer program developed by van Eck, NJ et al. for constructing and mapping bibliometric maps. It utilizes the VOS mapping technique to generate visualizations based on the co-occurrence matrix (Label view, Density view, Cluster density view, Scatter view). The program has the ability to handle large maps. In this paper, we use VOSviewer (1.6.20.0) to analyze the coupling among authors, countries, and journals [22].

Origin is a scientific mapping and data analysis software developed by Origin Lab. It possesses a variety of strengths, including its ability to process and analyze data, its user-friendly interface, its capacity to adapt to different user preferences, and its provision of multifunctional analysis tools. Additionally, it can generate high-quality 2D and 3D graphics. The objective is to create a trend chart that illustrates the publication years of articles. This was achieved by employing a co-occurrence diagram that demonstrated the cooperation between countries [23].

Tableau is a widely used visualization software that supports the use of multiple databases and the import of data in a variety of formats, thereby assisting users in creating a variety of charts for the purposes of visualization and data analysis. In this paper, we employed Tableau to create a map of the number of national communications [24].

## 3. Results

### 3.1. Basic Quantitative Information

**Figure 2:**
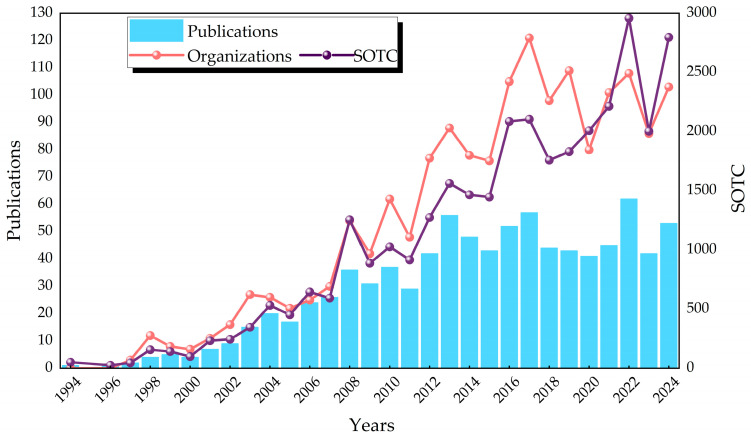
Trend chart of publication years of articles. Annual publications on *Dhc*, *Dhb*, *Dhgm*, participating organizations of the year, and summation of total citations.

The 899 papers utilized in this study originated from 2,511 authors from 750 institutions in 40 countries, were published in 142 journals, and cited 17,756 citations. A comprehensive review of the extant literature pertaining to the three organohalide-respiring bacteria reveals a general upward trend from 1994 to 2012, with a subsequent leveling off post-2012, accompanied by diminished variability; the fluctuation in the number of published papers has decreased, and the research field has shifted from the previous dynamic changes to a more stable stage. The increase in the number of publications was accompanied by a concomitant increase in the number of organizations and the summation of total citations (SOTCs) (Figure 2). This suggests that as the number of research results increases, the number of organizations involved in research increases as well, and their influence is strengthened. The number of papers published in 2022, as well as the sum of total citations, reached unprecedented levels. The second peak occurred in 2017, a year in which publications, citations, and organizations involved were all at higher values. Notably, three highly influential articles appeared in 2017, all with 100 or more citations [25,26,27].

### 3.2. Authors and Inter-Author Cooperation

A thorough analysis of the authors of the articles was conducted to ascertain the primary contributors to the written material and the specific areas of study that were most extensively explored by the most prominent authors. Lotka’s laws indicated that approximately 60% of the total number of authors in a given field have authored a single article. According to statistical data, there are 2511 authors engaged in the composition of articles, of which 1697 authors have authored a single paper, constituting approximately 67.58% of the total number, thereby aligning with Lotka’s laws.

In accordance with Price’s Law, he asserts that the number of core author publications in a given field should not be less than the product of 0.749 and the square root of the author’s most significant publication [28].

$$m=0.749\times \sqrt{n_{max}}$$

The highest number of publications among the authors enumerated was 66, $n_{max}$ equals 66, and the value of m has been calculated to be approximately 6.08, so those with more than 7 publications (including 7) are designated as “core authors,” for a total of 93.

The table illustrates the top five authors in terms of publications (Table 1), with Frank E. Löffler being the author with the most articles written and the highest average citation, and Lisa Alvarez-Cohen being the author with the highest H-index. The three authors, Frank E. Löffler, Elizabeth Anne Edwards, and Lorenz Adrian, co-authored a highly cited article introducing a new genus and species of *Dehalococcoides*. The article provides a detailed description of the strain’s morphology, dechlorination performance and conditions, as well as a comparative analysis of its gene sequences [29].

### 3.3. Country and Inter-Country Cooperation

**Figure 3:**
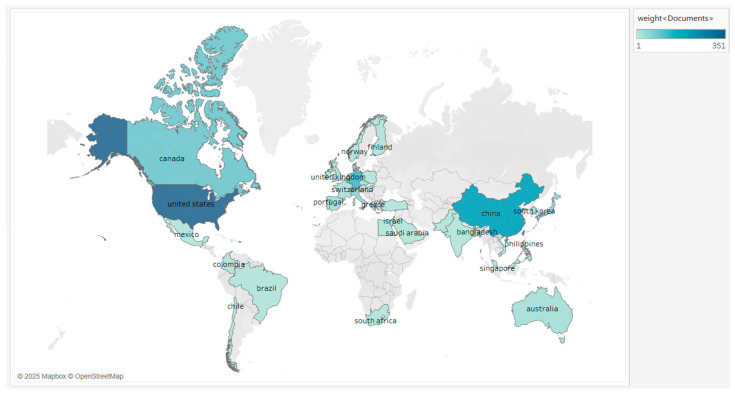
Map of the number of publications by country. The utilization of colors in this context serves to denote the quantity of publications from each nation. The intensity of the color, which is expressed as a darker shade, corresponds to the number of publications from a given nation.

The analysis of the country to which the paper belongs allows for studying the amount of national research contribution in the field, as well as the international cooperation and the time span of participation in the research. These papers originated from 40 countries around the world, and the top five countries in terms of number of publications are the USA, Germany, China, Canada, and Switzerland (Table 2 and Figure 3), with the USA ranking first in terms of number of publications and citations, and having the most collaborations with other countries. On a year-by-year basis (with ten years as the interval), the USA, Germany, and Canada as a whole maintain a high level of activity in all time periods, while China is more prominent in the later period, and the global research fervor in this area continues. In the initial phase (1994–2004), the number of countries contributing articles was relatively small, with a total of 68 articles from 11 countries, of which the USA, Germany, and Switzerland led the world with more than 10 articles. In the medium term (2005–2014), there was explosive growth in the number of publications, reaching a total of 346, with the USA leading the list with 178 papers, and it was observed that some countries, such as China and Singapore, joined the research in this field. The later period (2015–2024) shows a steady development, with a total of 482 articles in 36 participating countries during the decade, and China has jumped to become the country with the highest number of articles, while the USA and Germany are still at the top of the world. Although China ranks second in the world in terms of the total number of publications, the average citation is still relatively low, and the index of international cooperation is lower than that of Germany, suggesting that China should put more emphasis on international cooperation and high-impact papers in its future development.

**Figure 4:**
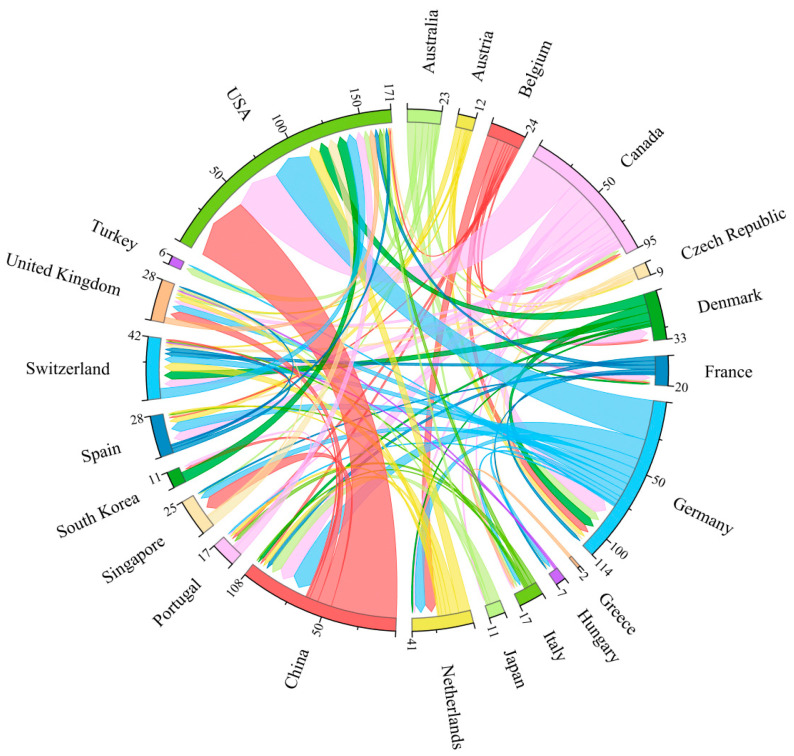
A co-occurrence diagram of cooperation between countries. A node represents a country, and the number of arcs in the outer circle indicates the extent of cooperation. The lines represent inter-country cooperation, and thicker lines indicate closer cooperation.

As illustrated by the inter-country cooperation co-linear graph, China and the USA have the closest cooperation, followed by Canada and the USA, and then Germany and the USA. This indicates that the three countries with the top three publication volumes have very close contact with each other and belong to the core of research cooperation. Concurrently, the top five countries with the highest publication volume are also the five countries with the closest international cooperation. Several European countries have demonstrated a strong collaborative spirit, evidenced by their cooperation with each other (e.g., Germany, Switzerland, and the United Kingdom) as well as with countries on other continents. This collaborative spirit is indicative of the synergistic nature of the European national research system (Figure 4).

### 3.4. Journals

Bradford’s Law states that if the number of papers in a certain discipline is ranked in a decreasing order, these journals can be divided into “core region” and other regions. Each region should correspond to a total number of papers that is approximately equal. The proportion of the number of journals in each region is approximately 1:n:n^2^. According to the content of Bradford’s law, we divided the 142 journals in which the articles were published into three regions: the core region, region 1, and region 2. Core region–region 1–region 2 = 1:4:4^2^, which conforms to the content of Bradford’s law [30]. The core journals are the top three journals in terms of the number of publications. «Environmental Science & Technology» is the journal with the highest number of publications, with a total of 160. It holds a certain leading position in the field of environmental science and technology and has a high impact factor. This status makes it more likely to attract authors to submit articles and use it for literature reviews. The journal with the highest citation frequency and average citation is «Applied and Environmental Microbiology», which far exceeds other journals in terms of citations, and it is highly regarded and influential. These journals are categorized as either Q1 (top 25% of the impact factor distribution) or Q2 (between the 25th and 50th percentiles) (Table 3).

### 3.5. Keyword Cluster Analysis and Burst Analysis

Keywords condense and summarize the core content of an article. Observing their frequency and time of occurrence can determine the hotspots and future frontiers of articles in this field. We mapped the top 50 keywords in terms of frequency and found that they fell into three clusters. The keywords that appeared most frequently were reductive dechlorination (400 times), tetrachloroethene (333 times), *Dehalococcoides* (287 times), vinyl chloride (260 times), and dechlorination (205 times). Reductive dechlorination is one of the central research themes in the field and runs throughout the study.

**Figure 5:**
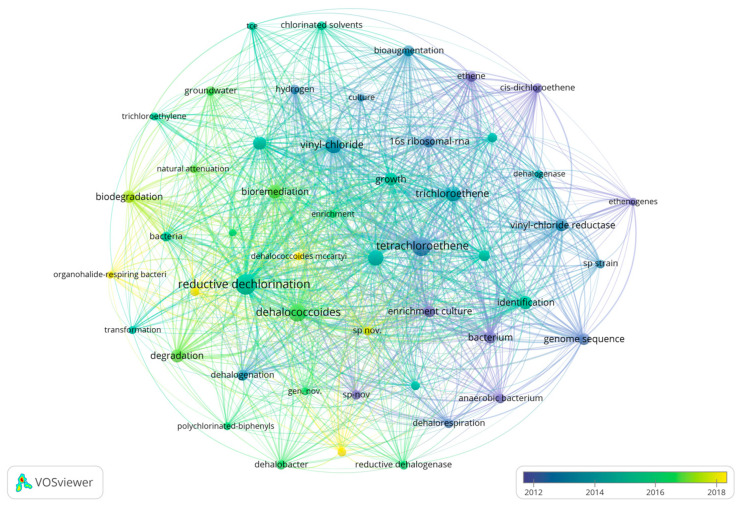
The keyword co-linear graph. For each node in the graph, a corresponding keyword exists, with node size corresponding to the occurrence frequency. Association among keywords occurs through edges connecting nodes, with thicker edges signifying more frequent or more intense occurrence relationships. Clusters or categories of keywords are differentiated by different colors, while color brightness indicates the relative time of occurrence—increasing brightness indicates more recent occurrence.

**Figure 6:**
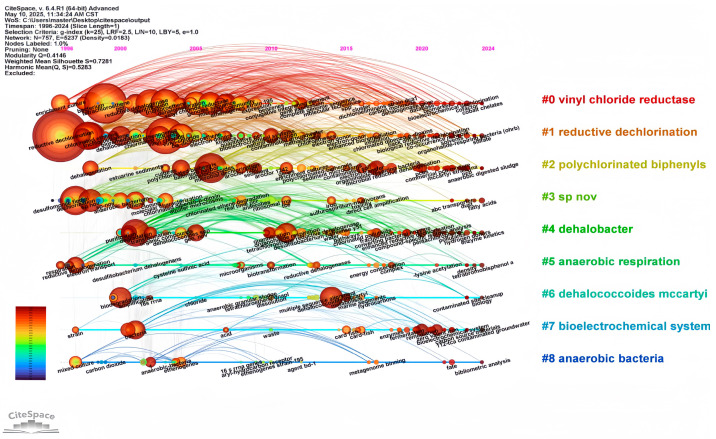
The keyword timeline graph. The graph utilizes diverse sizes and colors to represent different keywords. The images are plotted according to two criteria: the year of occurrence of the keywords and the closeness of the links with other keywords.

**Figure 7:**
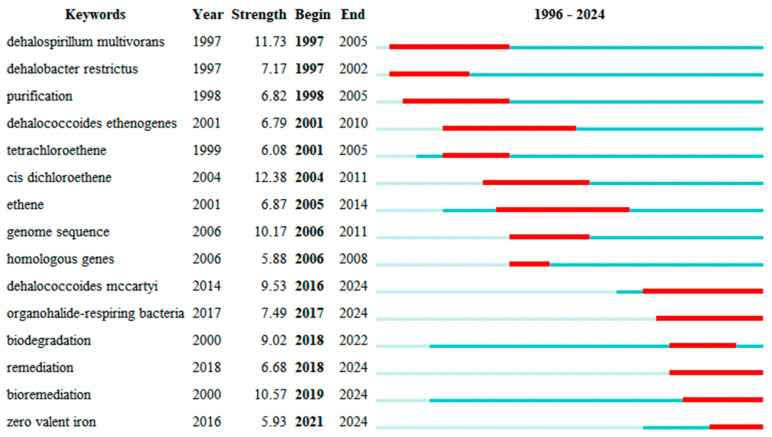
The keyword outbreak chart. The length of the color bar and the color shades represent the keywords in the corresponding year of research. The color bar’s length is indicative of the duration of the keyword’s presence in the data. The red bar indicates the period during which the keyword experienced an outbreak.

Various keywords represent various time frames, depicting changing research directions in this area. Generally, research interest has progressed gradually over time, becoming more specific. As shown in Figure 5, color shades represent the years in which keywords mainly appear. Figure 6 and Figure 7 are timeline pictures of keywords. Figure 6 plots one year as a slice to show dynamic research progress. The node size represents heat; the bigger the node, the more attention it receives. The connecting line represents node association; the thicker the line and the darker the color, the closer the association. Figure 7 shows the keywords on the left, the main appearance years in the middle, and a visual timeline graph on the right. The red line segment indicates the time period when the keywords receive the most attention. Integrating the three graphs shows that initially, research was more interested in discovering and isolating dehalogenating bacteria. This focus was subsequently supplemented by developments in genome sequencing and detection technology. More recently, research interest has focused on terms like organohalide-respiring bacteria or *Dehalococcoides*, with increasing attention to metabolic processes in specific microbial genera. Research interest has also moved to allied areas, including biodegradation, bioremediation, and more comprehensive environmental protection strategies. Materials like zero-valent iron, which couple dechlorination, have gradually received increasing attention in recent years, increasing the range of applications of the field in environmental as well as ecological remediation.

**Figure 8:**
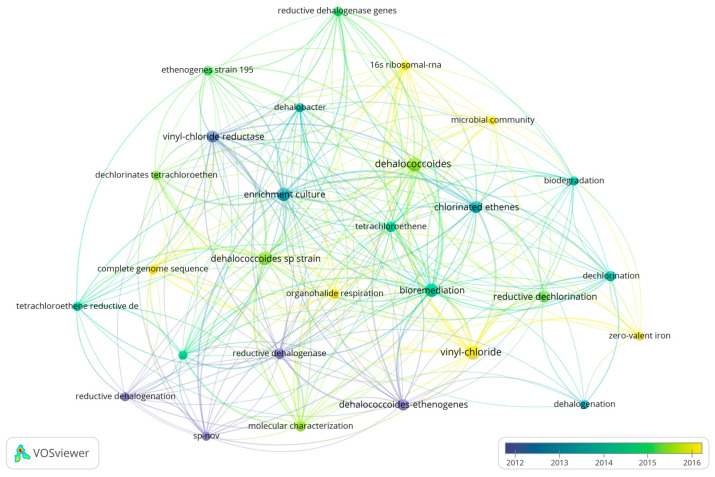
The keyword analysis chart of reviews. Other elements correspond to the annotations of Figure 5.

A total of 54 reviews, authored by 156 scholars involved in the project, were analyzed (Figure 8). Hauke Smidt’s contribution was found to be particularly significant, with four articles he authored being cited 484 times. The results of the keyword analysis of the review, and the keyword analysis of the literature system after combining the review and the article present a high degree of similarity.

## 4. Discussion

In this paper, a bibliometric survey of publications in the field of three categories of organohalide-respiring bacteria in the WOS database from 1994 to 2024 was carried out. A total of 899 data samples were obtained after manual screening, and the data were analyzed in terms of annual publication volume, authors, countries, journals, keywords, etc. We used this analysis to examine past research results and hot spots, as well as the future development trend in this field. A general upward trend in the number of publications was observed, accompanied by fluctuations in the number of participating organizations and the total number of citations.

A comprehensive data analysis reveals that the aggregate number of authors contributing to the study is 2511, with 93 core contributors. The author with the highest number of publications and the highest total citations is Frank E. Löffler. The authors collaborate closely, fostering a more stable group that promotes the development of the field. The study encompasses 40 countries, reflecting diverse international participation. The following three countries have been identified as the leading global contributors to publications: the USA, China, and Germany have established a close collaborative relationship with the aim of enhancing research contributions in this domain. The basis of this cooperation is the complementarity of resources, with countries such as the USA, Germany, and Canada providing experimental strains and equipment to support other nations [31,32].

A total of 142 journals are participating in the program, and three of these journals are considered of particular importance. The journals with the highest number of articles and the highest citation frequency are «Environmental Science & Technology» and «Applied and Environmental Microbiology». «Environmental Science & Technology», a leading journal in the field of the environment, is more likely to attract a significant number of researchers to submit high-quality manuscripts. The journal’s extensive coverage of the field and robust interdisciplinary approach lead to a greater number of published articles.

Through cluster analysis, timeline analysis, and burst analysis of publication keywords, it was found that past research has primarily focused on three types of organohalide-respiring bacteria as model microorganisms. The emphasis has been on the mechanisms of reductive dechlorination of pollutants such as organohalides, with the aim of analyzing microbial metabolic pathways, gene sequences, and environmental adaptability. Some high-frequency terms include “reductive dechlorination,” “tetrachloroethene,” and “*Dehalococcoides*.” However, as time progresses, the focus of research has shifted. From a technical perspective, the direction has evolved from initial screening and identification of microbial basics to macro-level technologies such as ecological restoration, gradually emphasizing the performance and efficacy of the three types of organohalide-respiring bacteria. In future research, due to the emergence of more complex and emerging pollutants, studies are likely to expand into areas such as combined pollution remediation, inter-species microbial interactions within the microbiome, and multidisciplinary technology coupling for pollution control.

1. Combined pollution remediation.

The majority of naturally contaminated sites are distinguished by the concurrent presence of multiple pollutants and the synergistic amplification of ecotoxicity. Despite the execution of several studies on the co-contamination of organohalogenides and metals [33], co-contamination of organic halides and arsenic [34], and complex contamination of chromium and organochlorine solvents [35], the transformation to real contaminated sites is still faced with a number of challenges. The future should therefore entail the dissolution of idealizations and the strengthening of long-term in situ testing of real sites. In the future, it is imperative to challenge the idealized research generalizations, fortify the long-term in situ experiments on actual sites, and methodically confront the intricacies posed by pollutants.

2. Inter-species microbial interactions within the microbiome.

A plethora of studies have previously centered on the genomic, transcriptomic, and proteomic analyses of individual organohalide-respiring bacteria [36,37]. These studies have established a substantial foundation for elucidating the metabolic mechanism of dechlorination within a singular colony. However, given the complexity inherent in microbial communities within natural ecological environments, it is often challenging to fully simulate the interaction network present in real habitats within the confines of a single colony study. Consequently, it is imperative to examine the interactions among various strains of microorganisms, including the dynamic response of the community function and the environmental impact factors. This comprehensive approach, encompassing the optimization of individual strains and the transition to community-level management, is essential for the effective mitigation of organohalogen pollution.

3. Multidisciplinary technology coupling for pollution control.

A number of studies have investigated the reductive dechlorination of zero-valent iron in conjunction with biostimulation [38,39,40], microplastics [41], and waste activated sludge [42]. These studies have demonstrated that altering the microbial community structure can enhance the enrichment of OHRB and stimulate its dechlorination. Furthermore, the combination of physicochemical modes (e.g., adsorption) and biodegradation has been shown to achieve dechlorination [43]. In order to achieve more efficient reductive dechlorination and environmental remediation, there is a need for further exploration of the coupling technology of new functional materials and microorganisms. In addition, the introduction of innovative dechlorination technologies from various disciplines is necessary to develop new pollution control strategies.

4. Further in-depth studies of omics technology.

In the domain of organohalogen remediation, omics technology has emerged as a pivotal instrument. In previous studies, researchers have extensively employed genomics, transcriptomics, and proteomics to sequence the genomes of organohalide-respiring bacteria and to mine key genes involved in the dehalogenation pathway. These studies have provided unprecedented insights into microbial communities and metabolic processes. In the future, the application of genomic technologies in pollution control is expected to expand in multiple directions, and the integration of multiple genomes will become increasingly important. The integration of data from multiple sources will facilitate the development of a more comprehensive and holistic understanding of microbial and pollutant interactions.

This study is predicated on bibliometrics and visualization analysis, employing software such as Citespace and VOSviewer to facilitate enhanced comprehension among researchers of future research trends in this field. Notwithstanding, the study is not without its limitations. The data used in this study were obtained exclusively from the WOS database, which may result in the omission of relevant research available in other academic databases. Although efforts were made to accurately screen authors and countries during the literature review, the possibility of software-related errors or other unforeseen issues cannot be entirely ruled out.

## 5. Conclusions

In this study, we employed the Citespace and VOSviewer software to meticulously and visually examine 899 articles on three types of specialized organohalide-respiring bacteria: *Dehalococcoides*, *Dehalobacter*, and *Dehalogenimonas*. These articles were sourced from the WOS database, spanning the period from 1994 to 2024. The main objective of this study was to trace back the research history of the three types of specialized organohalide-respiring bacteria and to provide references for future development trends. Over the past three decades, there has been an overall upward and then steady trend in the number of publications, with the United States dominating the early years, China increasing its influence in the later years, and close cooperation between countries. A total of 93 out of 2511 authors are core authors, and 3 out of 142 journals are core journals. In the initial stages of research on Keyword Clustering, the focus was on basic strains and substances. As the investigation progressed, it turned towards a more detailed exploration of microbial characteristics at the molecular level. In recent years, there has been a gradual transition towards the application of microbial and ecological remediation techniques. It is recommended that future research encompass a broad spectrum of disciplines. In the field of pollution remediation, the approach is not limited to a singular remediation technique. Instead, it integrates multi-faceted remediation methodologies to address contemporary and complex pollution scenarios. This integration is further augmented by the incorporating multi-omics technology, multi-scale monitoring methodologies, and an intelligent decision-making system. This integration serves to overcome the limitations of conventional remediation technologies, thereby facilitating the effective management of complex pollution situations.

Regarding the optimization of microbial interactions, the symbiotic, competitive and synergistic relationships between organohalide-respiring bacteria and other microbial communities will be explored more deeply. Moreover, relying on a single data source is considered a potential limitation. In the future, we will overcome the constraint of a single database and incorporate data from multiple databases, thereby promoting the innovative development of the research and application of organohalide-respiring bacteria.

## Figures and Tables

**Table 1:** A comprehensive overview of the documents, citations, average citation, and H-index of the top five authors from 1994 to 2024.

Rank	Author	Documents	Citations	Average Citation/Publication	H-Index
1	Frank E. Löffler	66	4980	75.45	48
2	Lorenz Adrian	52	3489	67.1	41
3	Jianzhong He	51	2151	42.18	39
4	Elizabeth Anne Edwards	50	2745	54.9	50
5	Lisa Alvarez-Cohen	38	1975	51.97	56

**Table 2:** The number of documents, the total number of citations, and the average citation for the ten countries with the highest number of publications from 1994 to 2024.

Rank	Country	Documents	Citations	Average Citation/ Publication
1	USA	351	18,616	53.04
2	China	202	4103	20.31
3	Germany	135	6862	50.83
4	Canada	89	4587	51.54
5	Singapore	52	1831	35.21
6	Italy	52	1430	27.5
7	Japan	51	1493	29.27
8	Netherlands	44	2962	67.32
9	Switzerland	41	2121	51.73
10	Denmark	29	1211	41.76

**Table 3:** Describes the number of documents, citations, average citations, impact factor, and category quartile of the top ten journals in terms of number of publications from 1994 to 2024.

Rank	Journal Name	Documents	Citations	Average Citation/Publication	Journal Impact Factor ™(Five Year)	Category Quartile
1	Environmental Science & Technology	160	6622	41.39	11.7	Q1
2	Applied and Environmental Microbiology	92	7857	85.4	4.6	Q2
3	Fems Microbiology Ecology	43	1251	29.09	4.2	Q2
4	Journal of Hazardous Materials	38	817	21.5	11.9	Q1
5	Frontiers in Microbiology	36	754	20.94	5.1	Q2
6	Water Research	32	1318	41.19	12.2	Q1
7	Science of the Total Environment	30	559	18.63	8.6	Q1
8	Chemosphere	30	791	26.37	7.7	Q1
9	Environmental Pollution	25	679	27.16	8.3	Q1
10	Journal of Contaminant Hydrology	23	638	27.74	3.4	Q2

## Data Availability

The original contributions presented in this study are included in the article. Further inquiries can be directed at the corresponding author.

## Supplementary Materials

The following supporting information can be downloaded at: https://www.mdpi.com/article/10.3390/microorganisms13071668/s1, Figure S1: PRISMA 2020 Flow Diagram for Systematic Review Based on Web of Science Core Collection.
