# Supplementary figures and images for: Bibliometrics and Visualization Analysis of Three Obligate Organohalide Respiring Bacteria Genera: A Systematic Review
Source: Microorganisms. 2025 Jul 16;13(7):1668. doi: 10.3390/microorganisms13071668 (PMC12298165; doi:10.3390/microorganisms13071668)

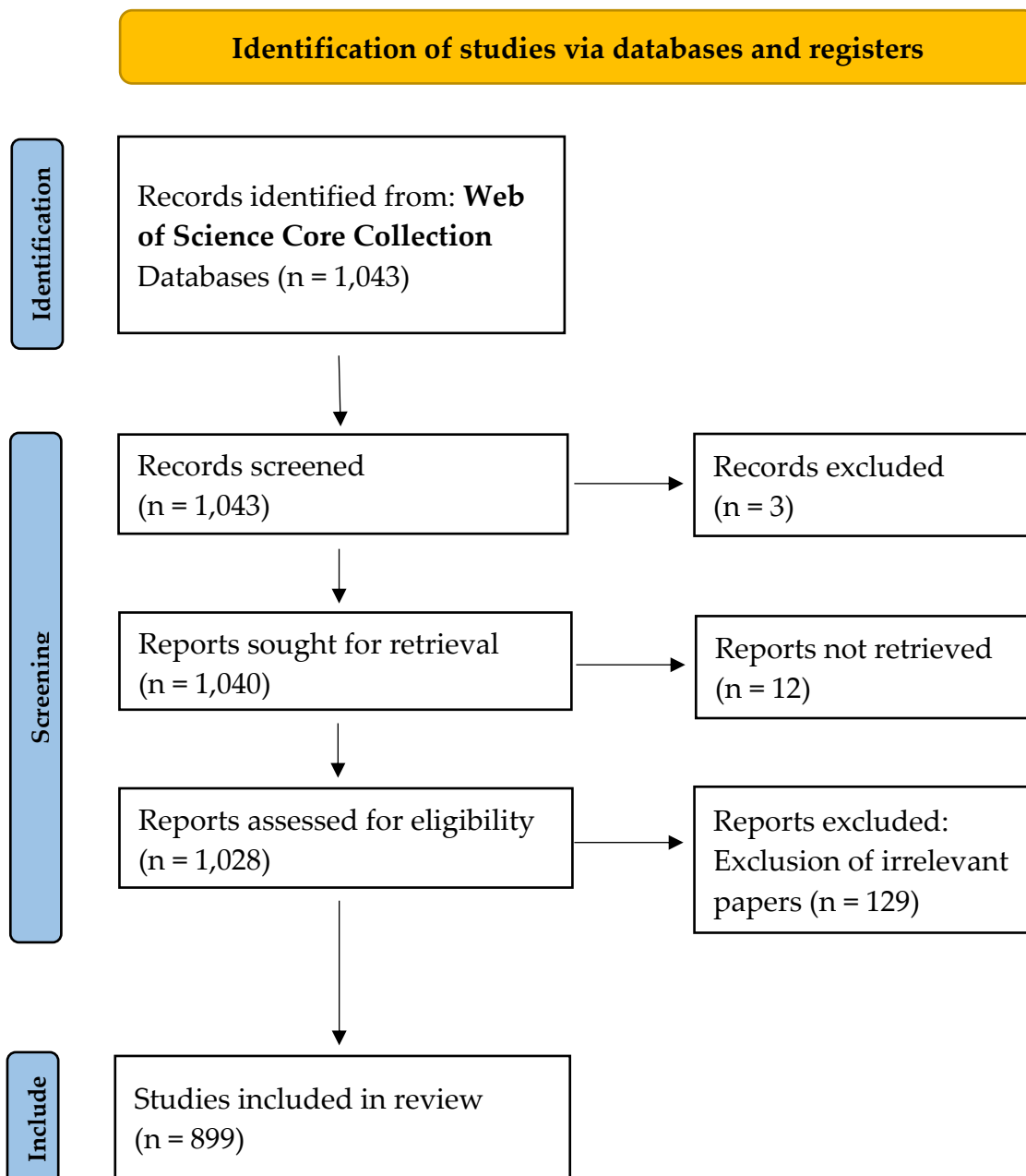

**Figure S1.** PRISMA 2020 flow diagram for systematic review based on Web of Science core collection.

Supplement: Supplementary file 1 [file microorganisms-13-01668-s001.zip › microorganisms-3714957-supplementary.pdf]
